# Predictors of Psychological Distress in Women with Endometriosis: The Role of Multimorbidity, Body Image, and Self-Criticism

**DOI:** 10.3390/ijerph18073453

**Published:** 2021-03-26

**Authors:** Shulamit Geller, Sigal Levy, Sapir Ashkeloni, Bar Roeh, Ensherah Sbiet, Ronit Avitsur

**Affiliations:** 1 School of Behavioral Sciences, The Academic College of Tel Aviv-Yaffo, Tel-Aviv 68182, Israel; sapirashkeloni@gmail.com (S.A.); barroeh@gmail.com (B.R.); ensherah.sbiet18@gmail.com (E.S.); avitsur@mta.ac.il (R.A.); 2 Statistical Education Unit, The Academic College of Tel Aviv-Yaffo, Tel-Aviv 68182, Israel; levy@mta.ac.il

**Keywords:** endometriosis, multimorbidity, body image, self-criticism, pain intensity

## Abstract

While large numbers of women report high levels of psychological distress associated with endometriosis, others report levels of distress that are comparable to those of healthy women. Thus, the aim of the current study was to develop an explanatory model for the effect of endometriosis on women’s psychological distress. Furthermore, it sought to further investigate the role of body image, self-criticism, and pain intensity on the psychological distress associated with endometriosis and establish the effect of chronic illness load on the development of this distress. This study comprised a total of 247 women aged 20–49 (*M* = 31.3, *SD* = 6.4)—73 suffering from endometriosis only, 62 suffering from endometriosis and an additional chronical illness (ACI), and 112 healthy peers (HP)—who completed the Patient Health Questionnaire, the Generalized Anxiety Disorder-Item Scale, the Body Appreciation Scale-2, and the Self-Criticism Sub-Scale. When comparing each endometriosis group to their HP’s, we found that the differences between HP and endometriosis ACI in depression and anxiety were mediated by body image (Betas = 0.17 and 0.09, respectively, *p*’s < 0.05) and self-criticism (Betas = 0.23 and 0.26, respectively, *p*’s < 0.05). When comparing endometriosis participants to endometriosis ACI participants, differences in depression were mediated by body image, self-criticism, and pain intensity (Betas = 0.12, 0.13, 0.13 respectively, *p*’s < 0.05), and the differences in anxiety were mediated by self-criticism and pain intensity (Betas = 0.19, 0.08, respectively, *p*’s < 0.05). Physicians and other health professionals are advised to detect women with endometriosis ACI who are distressed, and to offer them appropriate intervention.

## 1. Introduction

Endometriosis is an inflammatory disease associated with pelvic pain and infertility that is characterized by lesions of endometrial-like tissue outside of the uterus [1]. It is an inexplicable condition involving an uncertain and contested etiology [2] that affects approximately 6–10% of all woman of reproductive age [3,4]. Common symptoms are heavy and/or painful periods, pelvic pain, fatigue, congestive dysmenorrhea, heavy menstrual bleeding, and deep dyspareunia [5,6] as well as infertility [7,8].

The diagnosis and experience of the disease can involve a number of spheres of a woman’s life: emotional [9], marital, sexual [10,11], professional [12], and psychological (for review, see [13]) among others. For example, numerous studies have reported a significant incidence of anxiety and depressive symptoms among women with endometriosis that could influence the severity of symptoms and the health-related quality of their life [14,15,16]. Furthermore, distressed endometriosis patients, i.e., women with high levels of anxiety and depression, have been found to present an overall negative sense of female identity with lower self-esteem and poorer body image than non-distressed patients [17].

In spite of such potential significant allusions, there is a paucity of research on the link between endometriosis and psychological distress symptoms. Furthermore, while large numbers of women report high levels of psychological distress associated with endometriosis, others report levels that are similar to those of healthy women [17,18]. We wished to address this research gap by examining the link between endometriosis and distress symptoms and identifying the psychological mechanisms associated with this link. There is a need to focus on the individual differences, which seem to play an important role in mediating the psychological impact of endometriosis, in order to develop an explanatory model that describes how endometriosis affects women differently [19]. Such a model would assist professionals in the development and implementation of targeted multidisciplinary treatment strategies.

The increased risk of depression and anxiety in women suffering from endometriosis may be attributed, in part, to changes in their quality of life due to impaired body image [17,19]. Indeed, distressed body image was found to be associated with lower self-esteem and increased symptoms of depression and anxiety among endometriosis patients [17,19]. This link is supported by evidence indicating that women with endometriosis tend to be more concerned with their bodies than healthy individuals [20]. This may be due to the functional limitations and appearance changes associated with weight gain caused by hormonal therapy, scars from surgery, paleness resulting from heavy bleeding and anemia [21], or physical symptoms caused by induced menopause (tiredness, hot flashes, vaginal dryness, and poor libido) [19].

From a complementary sociocultural perspective, it may be argued that negative attitudes and concerns about the body experienced more by women with endometriosis involve self-perceptions of being negatively viewed and judged as, for example, unattractive or worthless [22] due to their distance from societal body ideals [23,24]. These negative self-evaluations, referred to here as self-criticism, constitute a maladaptive coping strategy with one’s body image attributes [25]. While self-criticism can be seen as an attempt to self-correct perceived defects or failures and thereby guarantee social approval and acceptance [26], it leads to an increase of negative affect [27] in the form of anxiety [28], depression, and depressive symptoms [29,30].

The presence of chronic and intense pain is a central symptom of endometriosis [31] and is considered a major stressor affecting quality of life [32] and causing psychological distress [33]. It is important to bear in mind that while psychological distress may modulate pain, it can also be a consequence of the pain [31]. As the prevalence of mood and anxiety disorders is greater among women who experience endometriosis-related pain than pain-free women [34], it is essential to identify pain intensity when assessing psychological distress among this population [35].

Previous research has suggested a comorbidity relationship between endometriosis and many other physical diseases. Although inconsistent, reports have posited a link between endometriosis and increased risk of migraines, diabetes mellitus, cardiovascular diseases, chronic liver disease, hypertension, hyperlipidemia, and autoimmune diseases such as systemic lupus erythematosus, rheumatoid arthritis, coeliac disease, multiple sclerosis, and inflammatory bowel disease [36,37,38,39]. Chronic illness is typically perceived as a severe stressor and often increases the risk of mood disorders. Studies have indicated that people with multimorbidity (i.e., the occurrence of two or more chronic medical conditions such as cancer, diabetes, heart disease and stroke [40] are approximately twice as likely to be depressed than people with only one chronic condition [41]. According to these findings, it is possible that the load of additional physical illnesses among vulnerable individuals suffering from endometriosis may further increase their risk of psychological distress and mood disorders.

The aim of the current study is to develop an explanatory model that demonstrates that psychological distress associated with endometriosis can be mediated by body image perceptions, self-criticism, and pain intensity. In addition, it explores the effect of having endometriosis and additional chronic illness (ACI) on the suggested mediation model.

The hypotheses of the study are: (1a) Differences will be found between healthy peers (HP), endometriosis only, and endometriosis ACI participants such that both endometriosis groups will present higher psychological distress, poorer body image, and greater self-criticism; (1b) Body image perception and self-criticism will mediate the association between health status (HP vs. endometriosis only participants and HP vs. endometriosis ACI participants) and psychological distress symptoms (depression and anxiety) among endometriosis participants; (2a) Pain intensity will be higher among endometriosis ACI participants than endometriosis only participants and; (2b) Body image perception, self-criticism, and pain intensity will mediate the link between having an additional chronic illness and psychological distress symptoms (depression and anxiety) among endometriosis patients.

## 2. Materials and Methods

This cross-sectional survey was carried out in Israel during 2020 as part of a research project on endometriosis and its association with psychological variables. Participants were recruited via two different methods: 1. Relevant forums over the internet—those who volunteered to participate in the study were given a link to a survey and asked to complete it electronically; 2. A snowball/convenience sample—students approached potential participants among their acquaintances, who were, in turn, asked to help expand the sample by recruiting more participants from their social networks in a multi-stage method. Individuals who agreed to take part in the study were sent a link to the survey and asked to complete it electronically.

**Figure 1:**
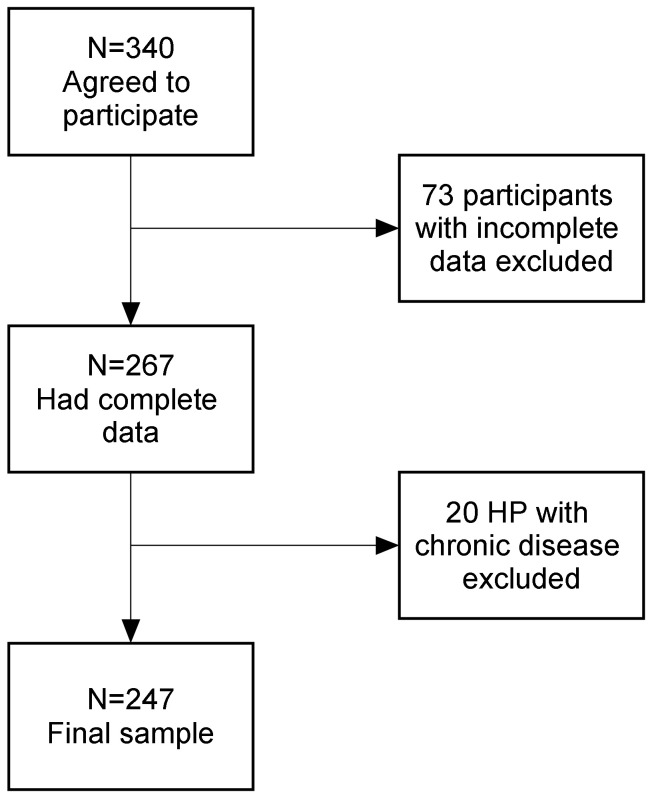
Data collection and recruitment.

Inclusion criteria were women aged 20–50, to cover average childbearing years, residing in Israel, and fluent in Hebrew to allow participation in the survey. Control and experimental groups were divided based on self-report of existing medical conditions. Diagnosis was not corroborated by medical records. A total of 247 women comprised the sample. Information about recruitment and data collection can be found in Figure 1. Women suffering from endometriosis were split into two groups: endometriosis only (73 women), and endometriosis ACI (62 women). The control group included 112 women who were HP. Participants were aged 20–49 (*M* = 31.3, *SD* = 6.4).

### 2.1. Measures

Participants reported their age, marital status, and number of children. In addition, they reported whether they are currently pregnant, as well as whether they are currently suffering from a chronic disease selected from a list of ACI (high blood pressure, diabetes, heart condition, asthma, irritable bowel disease, other). Women who reported being diagnosed with endometriosis also reported the time since their diagnosis, the time since first noticing endometriosis symptoms, and their age when first seeking medical help. They were asked to rate their endometriosis-related pain levels in the past month on a 7-point scale ranging from 1 (no pain) to 7 (unbearable pain).

Severity of depression was assessed using the 9-item Patient Health Questionnaire (PHQ-9) [42]. Participants rated items on a 4-point scale ranging from 0 (not at all) to 3 (nearly every day). The scores were added together to obtain a global score, which ranges from 0 to 27 with higher scores indicating higher levels of depression. Internal consistency of the PHQ-9 in the current study was satisfactory (Cronbach’s alpha = 0.89).

The Generalized Anxiety Disorder Scale (GAD-7) [43] is a 7-item self-report screening tool and severity measure for generalized anxiety (panic disorder, social anxiety disorder, and posttraumatic stress disorder). Participants rated items on a 4-point scale ranging from 0 (not at all) to 3 (nearly every day). The scores were added to obtain a total anxiety score, which ranges from 0 to 21 with higher scores indicating higher levels of anxiety. Internal consistency of the GAD-7 in the current study was satisfactory (Cronbach’s alpha = 0.93).

The Body Appreciation Scale-2 (BAS-2) [44,45] (Hebrew translation) is a 10-item measure that assesses acceptance of one’s body, respect and care for one’s body, and protection of one’s body from unrealistic beauty standards. All items were rated on a 5-point scale ranging from 1 (never) to 5 (always) and an overall score was computed as the mean of all items, with higher scores reflecting greater body appreciation and lower scores relating to distressed or poorer body image. Internal consistency of the BAS-2 in the current study was satisfactory (Cronbach’s alpha = 0.94).

Self-criticism (DEQ-SC) is a 23-item subscale of the Depressive Experiences Questionnaire (DEQ) [46] that reflects concern with failure and with being unable to meet high self-standards. Participants rated each item on a 7-point scale ranging from 1 (strongly disagree) to 7 (strongly agree). Scores were obtained by averaging across items. Higher scores reflect greater self-criticism. Internal consistency of the DEQ-SC in the current study was satisfactory (Cronbach’s alpha = 0.86).

### 2.2. Statistical Analysis

Descriptive statistics are presented as means, medians, ranges, and standard deviations or counts and percentages, as appropriate. Normality of the main study variables was assessed using the Wilks-Shapiro test. As some variables did not distribute normally, non-parametric tests were applied in addition to the parametric ones. Group comparisons in the main continuous outcome measures were done using one-way ANOVA with Tukey’s correction for multiple comparisons as well as the Kruskal-Wallis test, and in categorical variables using the chi-square test. The Pearson and Spearman correlation coefficients were used to evaluate the association between continuous variables. Process model 4 [47] was used to test the mediation hypotheses. Analysis was conducted using IBM SPSS v25, Process v3.4.

## 3. Results

Comparison of demographic data demonstrated that women suffering from endometriosis ACI were significantly older (*M* = 33.8, *Med* = 33, *R* = *20* − *48*, *SD* = 7.4) than women suffering from endometriosis only (*M* = 31.6, *Med* = *31*, *R* = *22* − *47*, *SD* = 5.8) or HP (*M* = 29.5, *Med* = *28*, *R* = *20* − *49*, *SD* = 5.5) (*F*(2, 230) = 9.7, *p* < 0.01). Of the total participants, 70% were in a relationship and 25% had children, with no significant differences between the groups in either measure.

Analysis of the correlations between background sociodemographic variables and psychological distress revealed no significant correlations. A comparison between the three groups in the main study variables is presented in Table 1 (Hypothesis 1a). We found that all groups differed from each other in depression, with depressive symptoms highest in the endometriosis ACI group and lowest in the HP group. Anxiety levels were also different between study groups, with levels significantly lower among HP than among endometriosis participants both with and without other diseases. The endometriosis ACI group reported lower body image and greater self-criticism than the two other groups.

Correlations between the main study variables, as well as their means and standard deviations for the entire sample, are presented in Table 2. We found significant correlations between all study variables (all *p*’s < 0.001).

### 3.1. Role of Body Image and Self-Criticism in the Emergence of Depressive Symptoms in Endometriosis Participants

**Figure 2:**
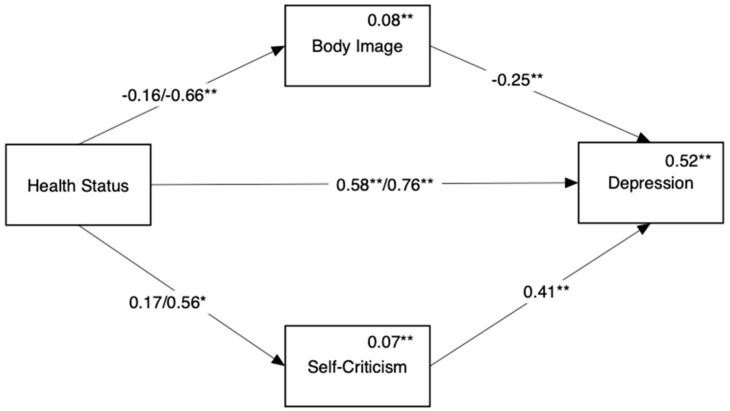
Mediation of the link between disease status and depression by body image and self-criticism. Reference group for comparison in the healthy controls. Numbers above the direct links are standardized regression coefficients. For paths originating from disease status, coefficients for endometriosis only appear on the left and coefficients for endometriosis ACI appear on the right. Numbers above variable names are multiple squared correlations. * *p* < 0.05, ** *p* < 0.01.

A mediation model was conducted for the three groups, with the HP group serving as the baseline comparison and the links to depression or anxiety mediated by body image and self-criticism. Figure 2 shows the mediation model predicting depression (Hypothesis 1b). We found a direct link between disease status and depression such that both endometriosis groups were significantly more depressed than the HP group. In addition, we found that the endometriosis ACI group differed from the HP group in depression through two indirect paths: body image (Beta = 0.17, 95% CI = [0.07, 0.29]) and self-criticism (Beta = 0.23, 95% CI = [0.10, 0.36]). No indirect paths were found when comparing the endometriosis only group with the HP group.

**Figure 3:**
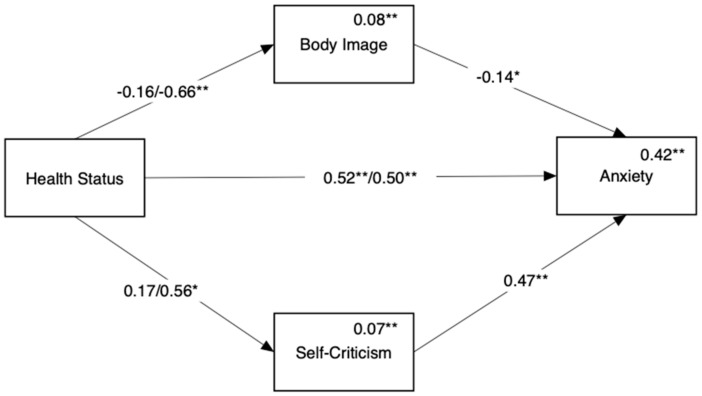
Mediation of the link between disease status and anxiety by body image and self-criticism. Reference group for comparison in the healthy controls. Numbers above the direct links are standardized regression coefficients. For paths originating from disease status, coefficients for endometriosis only appear on the left and coefficients for endometriosis ACI appear on the right. Numbers above variable names are multiple squared correlations. * *p* < 0.05, ** *p* < 0.01.

Figure 3 shows the mediation model predicting anxiety (Hypothesis 1b). We found a direct link between disease status and anxiety such that both endometriosis groups reported higher levels of anxiety symptoms compared to the HP group. In addition, we found that the endometriosis ACI group differed from the HP group in anxiety through two indirect paths: poorer body image (Beta = 0.09, 95% CI = [0.00, 0.20]) and higher self-criticism (Beta = 0.26, 95% CI = [0.11, 0.42]). No indirect paths were found when comparing the endometriosis only group with the HP group.

### 3.2. Role of Body Image and Self-Criticism in the Emergence of Depressive Symptoms in Endometriosis Participants with or without ACI

On comparing the endometriosis only and endometriosis ACI groups (Hypothesis 2a), we found a significant difference (*F*(*1132*) = 4.8, *p* < 0.05) in pain intensity during the month prior to participating in the study, with the former (*M* = 4.7, *Med* = *5.0*, *R* = *1.0* − *7.0*, *SD* = 1.9) suffering from higher pain intensity that the latter (*M* = 5.3, *Med* = *5.0*, *R* = *2.0* − *7.0*, *SD* = 1.4). A comparison of other endometriosis-related background measures (time since diagnosis, time since first noticing symptoms, age when first seeking medical help) showed no differences between the two endometriosis groups.

**Figure 4:**
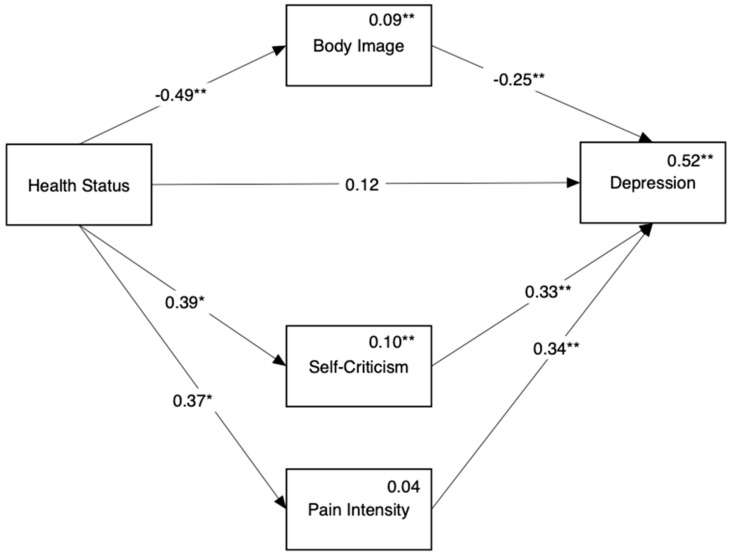
Mediation of the link between disease status (endometriosis only or endometriosis ACI) and depression by body image, self-criticism, and pain intensity. Reference group for comparison is the endometriosis only group. Numbers above the direct links are standardized regression coefficients. Numbers above variable names are multiple squared correlations. Age was included in the model as a covariate but is not presented in this figure. * *p* < 0.05, ** *p* < 0.01.

Figure 4 shows the mediation model predicting depression (Hypothesis 2b). We found no direct path from disease status to depression, yet we found three indirect paths between them: through body image (Beta = 0.12, 95% CI = [0.03, 0.25]), self-criticism (Beta = 0.13, 95% CI = [0.02, 0.25]), and pain intensity (Beta = 0.13, 95% CI = [0.01, 0.27]).

**Figure 5:**
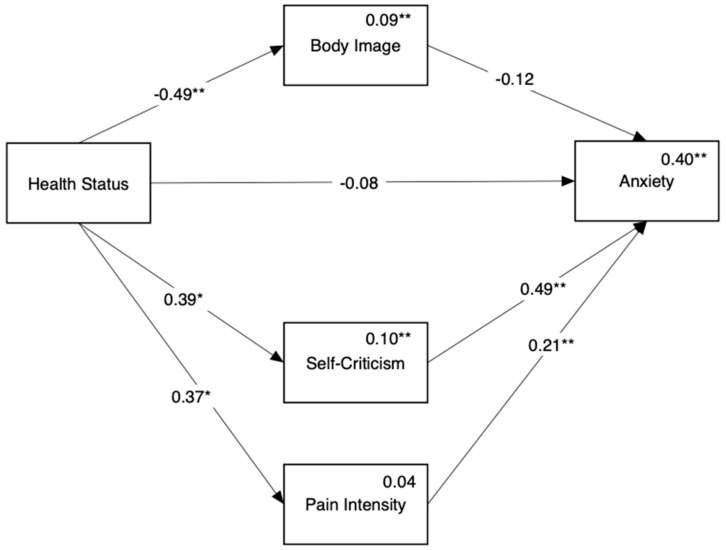
Mediation of the link between disease-status (endometriosis only or endometriosis ACI) and anxiety, by body image, self-criticism, and pain intensity. Reference group for comparison is the Endometriosis only group. Numbers above the direct links are standardized regression coefficients. Numbers above variable names are multiple squared correlations. Age was included in the model as a covariate, yet is not presented in this figure. * *p* < 0.05, ** *p* < 0.01.

Figure 5 shows the mediation model predicting anxiety (Hypothesis 2b). We found no direct path from disease status to anxiety, yet we found two indirect paths between them: through self-criticism (Beta = 0.19, 95% CI = [0.03, 0.37]) and pain intensity (Beta = 0.08, 95% CI = [0.002, 0.18]).

## 4. Discussion

This cross-sectional study aimed to develop an explanatory model for the effect of endometriosis on women’s psychological distress. More specifically, it sought to further investigate the role of body image, self-criticism, and pain intensity on the psychological distress associated with endometriosis and establish the effect of chronic illness load on the development of this distress.

In line with past reports [17,48], the current study demonstrated higher levels of depression and anxiety among women suffering from endometriosis than among HP. Furthermore, in accordance with previous findings on other illnesses, depression was higher among women suffering from endometriosis ACI than those suffering from endometriosis only [41,49]. These findings can be seen to account for the specific challenges faced by these women which generate anxiety and distress, such as uncertainty related to the cyclical and unpredictable manner of the disease [50]. The current study attempted to obtain a deeper understanding of the underlying mechanisms creating these multimorbidity-related differences. We found that endometriosis participants with an additional chronic illness tended to suffer from poorer body image and greater self-criticism than HP, which, in turn, led to more psychological distress. These findings are likely due to their acknowledgement that a sick body will constantly fail to meet societal standards of beauty [23]. This constant self-judgement may promote more feelings of failure and of the need to escape and thus perpetuates the vicious cycle of body shame and self-criticism [22]. Lastly, while COVID-19 has been found to associate with a notable increase in psychological distress worldwide [51], women with endometriosis suffered additional distress [52]. The mandatory self-isolation may have imposed negative psychological effects, which patients with endometriosis are more prone to. Furthermore, new obstacles emerged due to limited access to healthcare appointments, ultrasound evaluations, and surgeries [52]. It is possible that this additional burden added to the distress of the participants in our study, so that the level of psychological distress they reported was higher than it would be otherwise.

A comparison of disease-related factors between participants suffering from endometriosis only and participants suffering from endometriosis ACI revealed that in addition to poorer body image and greater self-criticism, pain intensity, which is a strong characteristic of endometriosis, was higher among the latter. The differences in depression between these two groups were fully mediated by body image, self-criticism, and pain intensity. The differences in anxiety between the two groups were also fully mediated by self-criticism and pain intensity but not by body image.

These findings add to the growing number of studies suggesting that not all women with endometriosis are necessarily more distressed than healthy women [17,18,53]. It may not be endometriosis per se that is responsible for depression and anxiety but rather the experience of comorbidities that do not merely produce an additive burden but interact and, in some instances, result in magnified effects [49]. This direction may be supported by the findings of Urteaga et al. [54], who used patient-generated health data and data-driven phenotyping, based mainly on reported signs and symptoms, to characterize four subtypes of endometriosis patients. Specifically, they described Phenotype A as a particularly severe endometriosis subtype with symptoms related to several comorbidities such as anxiety, depression, and other mood disorders, migraines, high blood pressure, and chronic fatigue syndrome. Based on this, future studies should focus on psychological distress among Phenotype A endometriosis women.

Multimorbidity and depression covary in a dose-dependent way such that a growing number of comorbidities are associated with a greater likelihood of depression and other mental health conditions [55]. In addition, it has now been established that the relationship between the incidence of physical diseases and depression is likely to be temporally bidirectional. Epidemiological research consistently shows that multimorbidity and depression often coexist; their copresence seems to trigger a cascade of disturbances that culminates in even greater strain in terms of disability and mortality (e.g., [56]). Recent research has detected a variety of biological (e.g., inflammation), psychosocial (e.g., stressful life events), and care-related drivers (e.g., polypharmacy) that possibly regulate the transition from multimorbidity to depression and vice versa [57]. There is evidence that the physical and psychic pain resulting from endometriosis is responsible for depression and that one disease aggravates the other. As a result, there is no consensus on the temporal issue when defining which is the preexisting condition [58]. A mutual relationship may be established, in which anxiety and depression increase pain perception and pain, in turn, can compromise depression in a vicious cycle manner [34].

Finally, the relationship between endometriosis and the above three predictors—body image, self-criticism, and pain intensity—may be conceptualized as a complex mutual interaction rather than a unidirectional causal link. For example, it may be hypothesized that while endometriosis and chronic pain are capable of conveying a negative body image [20], the adoption of a critical and self-deprecating attitude may increase maladaptive defensive responses focused on body image shame [59]. At the same time, it should be considered that psychological distress can amplify pain symptoms both emotionally and cognitively [60] as well as body image concerns and self-criticism [17]. Further studies are needed in order to investigate these aspects.

The absence of difference in body image between women with endometriosis only and HP in the current study may suggest that, despite its side effects, endometriosis only is not experienced as a salient visible condition and is therefore less affected by social stigma and negative stereotypes regarding the body [61]. A second possible explanation relates to the prevalence of body image concerns in western societies. Epidemiological studies have consistently shown that many younger women and men are at least moderately dissatisfied with their body weight or shape [62]—a phenomenon that has been related to the term “normative discontent” [63]. Thus, even though endometriosis may bear some degree of distressed body image, this might be obscured by the prevalence of normative discontent.

Based on our findings, future studies should focus on developing unique psychological interventions for women suffering from endometriosis, paying special attention to women suffering from multimorbidity. Targeting this chronic illness population, which has been shown to suffer from increased body image shame and self-criticism and to be prone to the shame–self-criticism vicious cycle [22,57], may help reduce their symptoms of depression and anxiety and the negative impacts of endometriosis (e.g., shame, feelings of inferiority). We hope that such studies will contribute to the development of interventions and coping strategies, such as compassionate mind training (CMT) [64] or psychological acceptance therapy [65]. Focusing on improving body image and alleviating self-criticism will likely improve body perception and assist women in coping with chronic illnesses.

The primary limitation of the current study is its cross-sectional design which constrains causal conclusions. Our promising path analytic findings should encourage the design of longitudinal intervention studies that investigate body image, self-criticism, and pain perception in women suffering from endometriosis, either with or without ACI. A second limitation concerns the validity of the model, which might be increased by controlling the severity and type of endometriosis and including the specific characteristics of pain symptoms related to endometriosis (e.g., [35]). Third, as the present findings suggest that the relationship between all predictors are complex and not unidirectional, more research is needed to confirm these mutual interactions between all predictors and psychological distress among endometriosis ACI women. Fourth, illness status was reported by our participants and their health status was not directly assessed. An additional examination by a medical professional would provide direct information for the association between illness and psychological distress. Fifth, participants were recruited online, and as a previous study showed that method of recruitment may affect the response of women with endometriosis [66], this study should be repeated using other recruitment methods. Sixth, the study was conducted during the COVID 19 pandemic, thus it is possible that it influenced the observed results. Finally, all of our participants were Hebrew-speaking; the application to Israeli minorities or non-Hebrew-speaking populations may therefore need further evaluation. Furthermore, there is no available information about the reproducibility and validity of the Hebrew version of the questionnaires that we used.

## 5. Conclusions

To conclude, the hypothesis that women with endometriosis present higher levels of depression and anxiety compared to HP has been confirmed by our study. A novel important finding is the highlighting that suffering from an additional chronic disease puts endometriosis patients at a greater risk for increased psychological distress. We also conclude that psychological distress may derive from concerns regarding body image, self-criticism, and pain intensity. Following our conclusions physicians and other health care professionals need to be guided to detect symptoms of anxiety and depression in women suffering from endometriosis ACI, and to deliver tailored counseling on the basis of patient characteristics.

## Figures and Tables

**Table 1:** Group comparison.

	HP (*n* = 112)		Endometriosis (*n* = 73)		Endometriosis ACI (*n* = 62)		
	M (SD)	Med, R	M (SD)	Med, R	M (SD)	Med, R	F(2, 244)
Depression	6.8 (5.0) ^a^	6.0, 0.0–24.0	10.9 (6.4) ^b^	10.0, 0.0–27.0	13.6 (5.9) ^c^	13.0, 0.0–25.0	30.6 **
Anxiety	5.9 (4.8) ^a^	4.0, 0.0–20.0	9.6 (6.4) ^b^	8.0, 0.0–21.0	10.9 (5.8) ^b^	10.0, 0.0–21.0	18.7 **
Body image	3.7 (0.8) ^a^	3.7, 1.7–5.0	3.6 (0.8) ^a^	3.7, 1.8–5.0	3.2 (0.9) ^b^	3.2, 1.4–5.0	7.1 **
Self-criticism	4.1 (0.8) ^a^	4.0, 2.5–6.0	4.2 (0.9) ^a^	4.1, 2.6–6.2	4.5 (0.9) ^b^	4.5, 2.2–6.7	4.1 *

ACI = additional chronic illness; HP = healthy peers; ^a,b,c^ Groups with similar indices do not differ at 0.05 significance level according to Tukey’s test for multiple comparisons. * *p* < 0.05, ** *p* < 0.01.

**Table 2:** Correlations between the study variables (all *p*’s < 0.001), means, and standard deviations.

	Self-Criticism	Depression	Anxiety	M (SD)
Body image	−0.54	−0.51	−0.39	3.5 (0.8)
Self-criticism		0.58	0.55	4.2 (0.9)
Depression			0.77	9.7 (6.3)
Anxiety				8.3 (6.0)

Because of the age differences between groups, age was included as a covariate in all mediation models.

## Data Availability

The datasets used and analyzed in the current study are available on request from the corresponding author.

## References

[1] Johnson N.P., Hummelshoj L., Adamson G.D., Keckstein J., Taylor H.S., Abrao M.S., Bush D., Kiesel L., Tamimi R., Sharpe-Timms K.L. (2017). World Endometriosis Society consensus on the classification of endometriosis. Hum. Reprod..

[2] Painter J.N., Anderson C.A., Nyholt D.R., Macgregor S., Li J., Lee S.H., Lambert A., Zhao Z.Z., Roseman F., Guo Q. (2011). Genome-wide association study identifies a locus at 7p15. 2 associated with endometriosis. Nat. Genet..

[3] Bulletti C., Coccia M.E., Battistoni S., Borini A. (2010). Endometriosis and infertility. J. Assist. Reprod. Genet..

[4] Kuznetsov L., Dworzynski K., Davies M., Overton C. (2017). Guideline committee diagnosis and management of endometriosis: Summary of NICE guidance. BMJ.

[5] Lemaire G. (2004). More than just menstrual cramps: Symptoms and uncertainty among women with endometriosis. J. Obstet. Gynecol. Neonatal Nurs..

[6] De Nardi P., Ferrari S. (2011). Deep Pelvic Endometriosis: A Multidisciplinary Approach.

[7] Meuleman C., Vandenabeele B., Fieuws S., Spiessens C., Timmerman D., D’Hooghe T. (2009). High prevalence of endometriosis in infertile women with normal ovulation and normospermic partners. Fertil. Steril..

[8] Pope C.J., Sharma V., Sharma S., Mazmanian D. (2015). A systematic review of the association between psychiatric disturbances and endometriosis. J. Obstet. Gynaecol. Can..

[9] Thomas E., Moss-Morris R., Faquhar C. (2006). Coping with emotions and abuse history in women with chronic pelvic pain. J. Psychosom. Res..

[10] Hudson N., Culley L., Law C., Mitchell H., Denny E., Raine-Fenning N. (2016). “We needed to change the mission statement of the marriage”: Biographical disruptions, appraisals and revisions among couples living with endometriosis. Sociol. Health Illn..

[11] Fritzer N., Haas D., Oppelt P., Renner S., Hornung D., Wölfler M., Ulrich U., Fischerlehner G., Sillem M., Hudelist G. (2013). More than just bad sex: Sexual dysfunction and distress in patients with endometriosis. Eur. J. Obstet. Gynecol. Reprod. Biol..

[12] Hansen K.E., Kesmodel U.S., Baldursson E.B., Schultz R., Forman A. (2013). The influence of endometriosis-related symptoms on work life and work ability: A study of Danish endometriosis patients in employment. Eur. J. Obstet. Gynecol. Reprod. Biol..

[13] Donatti L., Ramos D.G., Andres M.D.P., Passman L.J., Podgaec S. (2017). Patients with endometriosis using positive coping strategies have less depression, stress and pelvic pain. Einstein.

[14] Jia S.Z., Leng J.H., Shi J.H., Sun P.R., Lang J.H. (2012). Health-related quality of life in women with endometriosis: A systematic review. J. Ovarian Res..

[15] Friedl F., Riedl D., Fessler S., Wildt L., Walter M., Richter R., Schüßler G., Böttcher B. (2015). Impact of endometriosis on quality of life, anxiety, and depression: An Austrian perspective. Arch. Gynecol. Obstet..

[16] Vitale S.G., La Rosa V.L., Rapisarda A.M., Laganà A.S. (2016). Impact of endometriosis on quality of life and psychological well-being. J. Psychosom. Obstet. Gynaecol..

[17] Facchin F., Barbara G., Dridi D., Alberico D., Buggio L., Somigliana E., Saita E., Vercellini P. (2017). Mental health in women with endometriosis: Searching for predictors of psychological distress. Hum. Reprod..

[18] Facchin F., Barbara G., Saita E., Mosconi P., Roberto A., Fedele L., Vercellini P. (2015). Impact of endometriosis on quality of life and mental health: Pelvic pain makes the difference. J. Psychosom. Obstet. Gynecol..

[19] Facchin F., Saita E., Barbara G., Dridi D., Vercellini P. (2018). “Free butterflies will come out of these deep wounds”: A grounded theory of how endometriosis affects women’s psychological health. J. Health Psychol..

[20] Melis I., Litta P., Nappi L., Agus M., Melis G.B., Angioni S. (2014). Sexual function in women with deep endometriosis: Correlation with quality of life, intensity of pain, depression, anxiety, and body image. Int. J. Sex. Health.

[21] Moradi M., Parker M., Sneddon A., Lopez V., Ellwood D. (2014). Impact of endometriosis on women’s lives: A qualitative study. BMC Womens Health.

[22] Gilbert P., Gilbert P., Miles J. (2002). Body shame: A biopsychosocial conceptualisation and overview, with treatment implications. Body Shame Conceptualisation, Research and Treatment.

[23] Tiggemann M., Cash T.F., Smolak L. (2011). Sociocultural perspectives on human appearance and body image. Body Image: A Handbook of Science, Practice, and Prevention.

[24] Quick V. (2013). Social theory applied to body image and chronic illness in youth. Am. J. Lifestyle Med..

[25] Duarte C., Pinto-Gouveia J., Ferreira C., Batista D. (2015). Body image as a source of shame: A new measure for the assessment of the multifaceted nature of body image shame. Clin. Psychol. Psychother..

[26] Gilbert P. (2010). Compassion Focused Therapy: The CBT Distinctive Features Series.

[27] Shahar G. (2015). Erosion: The Psychopathology of Self-Criticism.

[28] Gilbert P., McEwan K., Mitra R., Franks L., Richter A., Rockliff H. (2008). Feeling safe and content: A specific affect regulation system? Relationship to depression, anxiety, stress, and self-criticism. J. Posit. Psychol..

[29] Campos R.C., Besser A., Blatt S.J. (2010). The mediating role of self-criticism and dependency in the association between perceptions of maternal caring and depressive symptoms. Depress. Anxiety.

[30] Joeng J.R., Turner S.L. (2015). Mediators between self-criticism and depression: Fear of compassion, self-compassion, and importance to others. J. Couns. Psychol..

[31] Morotti M., Vincent K., Becker C.M. (2017). Mechanisms of pain in endometriosis. Eur. J. Obstet. Gynecol. Reprod. Biol..

[32] Grogan S., Turley E., Cole J. (2018). “So many women suffer in silence”: A thematic analysis of women’s written accounts of coping with endometriosis. Psychol. Health.

[33] Zarbo C., Brugnera A., Frigerio L., Malandrino C., Rabboni M., Bondi E., Compare A. (2018). Behavioral, cognitive, and emotional coping strategies of women with endometriosis: A critical narrative review. Arch. Womens Ment. Health.

[34] Cavaggioni G., Lia C., Resta S., Antonielli T., Benedetti Panici P., Megiorni F., Porpora M.G. (2014). Are mood and anxiety disorders and alexithymia associated with endometriosis? A preliminary study. Biomed. Res. Int..

[35] Márki G., Bokor A., Rigó J., Rigó A. (2017). Physical pain and emotion regulation as the main predictive factors of health-related quality of life in women living with endometriosis. Hum. Reprod..

[36] Teng S.W., Horng H.C., Ho C.H., Yen M.S., Chao H.T., Wang P.H., Taiwan Association of Gynecology Systematic Review Group (2016). Women with endometriosis have higher comorbidities: Analysis of domestic data in Taiwan. J. Chin. Med. Assoc..

[37] Miller J.A., Missmer S.A., Vitonis A.F., Sarda V., Laufer M.R., DiVasta A.D. (2018). Prevalence of migraines in adolescents with endometriosis. Fertil. Steril..

[38] Schomacker M.L., Hansen K.E., Ramlau-Hansen C.H., Forman A. (2018). Is endometriosis associated with irritable bowel syndrome? A cross-sectional study. Eur. J. Obst. Gynecol. Reprod. Biol..

[39] Shigesi N., Kvaskoff M., Kirtley S., Feng Q., Fang H., Knight J.C., Missmer S.A., Rahmioglu N., Zondervan K.T., Becker C.M. (2019). The association between endometriosis and autoimmune diseases: A systematic review and meta-analysis. Hum. Reprod. Update.

[40] Van den Bussche H., Koller D., Kolonko T., Hansen H., Wegscheider K., Glaeske G., von Leitner E.C., Schäfer I., Schön G. (2011). Which chronic diseases and disease combinations are specific to multimorbidity in the elderly? Results of a claims data based cross-sectional study in Germany. BMC Public Health.

[41] Read J.R., Sharpe L., Modini M., Dear B.F. (2017). Multimorbidity and depression: A systematic review and meta-analysis. J. Affect. Disord..

[42] Kroenke K., Spitzer R.L., Williams J.B. (2001). The PHQ-9: Validity of a brief depression severity measure. J. Gen. Intern. Med..

[43] Spitzer R.L., Kroenke K., Williams J.B., Löwe B. (2006). A brief measure for assessing generalized anxiety disorder: The GAD-7. Arch. Intern. Med..

[44] Tylka T.L., Wood-Barcalow N.L. (2015). The Body Appreciation Scale-2: Item refinement and psychometric evaluation. Body Image.

[45] Geller S., Handelzalts J.E., Levy S., Boxer N., Todd J., Swami V. (2020). An examination of the factor structure and preliminary assessment of the psychometric properties of a Hebrew translation of the Body Appreciation Scale-2 (BAS-2). Body Image.

[46] Blatt S.J., D’Afflitti J.P., Quinlan D.M. (1976). Experiences of depression in normal young adults. J. Abnorm. Psychol..

[47] Hayes A.F. (2018). Partial, conditional, and moderated moderated mediation: Quantification, inference, and interpretation. Commun. Monogr..

[48] Laganà A.S., La Rosa V.L., Rapisarda A.M.C., Valenti G., Sapia F., Chiofalo B., Rossetti D., Ban Frangež H., Vrtačnik Bokal E., Vitale S.G. (2017). Anxiety and depression in patients with endometriosis: Impact and management challenges. Int. J. Womens Health.

[49] Birk J.L., Kronish M., Moise N., Falzon L., Yoon S., Davidson K.W. (2019). Depression and multimorbidity: Considering temporal characteristics of the associations between depression and multiple chronic diseases. Health Psychol..

[50] Denny E. (2009). “I never know from one day to another how I will feel”: Pain and uncertainty in women with endometriosis. Qual. Health Res..

[51] Torales J., O’Higgins M., Castaldelli-Maia J.M., Ventriglio A. (2020). The outbreak ofCOVID-19 coronavirus and its impact on global mental health. Int. J. Soc. Psychiatry.

[52] Leonardi M., Horne A.W., Vincent K., Sinclair J., Sherman K.A., Ciccia D., Condous G., Johnson N.P., Armour M. (2020). Self-management strategies to consider to combat endometriosis symptoms during the COVID-19 pandemic. Hum. Reprod. Open.

[53] Novais R.F.S.R., da Câmara-França B.E., Lasmar R.B., Lasmar B.P. (2018). Endometriosis and its relationship with depression. Int. J. Clin. Med..

[54] Urteaga I., McKillop M., Elhadad N. (2020). Learning endometriosis phenotypes from patient-generated data. NPJ Digit. Med..

[55] Barnett K., Mercer S.W., Norbury M., Watt G., Wyke S., Guthrie B. (2012). Epidemiology of multimorbidity and implications for health care, research, and medical education: A cross-sectional study. Lancet.

[56] Koyanagi A., Kohler-Forsberg O., Benros M.E., Munk Laursen T., Haro J.M., Nordentoft M., Hjorthoj C. (2018). Mortality in unipolar depression preceding and following chronic somatic diseases. Acta Psychiatr. Scand..

[57] Triolo F., Harber-Aschan L., Belvederi M.M., Calderón-Larranaga A., Vetrano D.L., Sjöberg L., Marengoni A., Dekhtyar S. (2020). The complex interplay between depression and multimorbidity in late life: Risks and pathways: Depression and multimorbidity. Mech. Ageing Dev..

[58] Lorenzatto C., Vieira M.J.N., Marques A., Benetti-Pinto C.L., Petta C.A. (2007). Evaluation of pain and depression in women with endometriosis after multiprofessional group intervention. Rev. Assoc. Med. Bras..

[59] Ferreira C., Dias B., Oliveira S. (2019). Behind women’s body image-focused shame: Exploring the role of fears of compassion and self-criticism. Eat. Behav..

[60] Sepulcri Rde P., do Amaral V.F. (2009). Depressive symptoms, anxiety, and quality of life in women with pelvic endometriosis. Eur. J. Obstet. Gynecol. Reprod. Biol..

[61] Eisenberg M.E., Neumark-Sztainer D., Haines J., Wall M., Neumark-Sztainer D., Falkner N., Little R.J.A. (2006). Weight-teasing and emotional well-being in adolescents: Longitudinal findings from Project EAT. J. Adolesc. Health.

[62] Grogan S. (2016). Body Image: Understanding Body Dissatisfaction in Men, Women and Children.

[63] Rodin J., Silberstein L., Striegel-Moore R., Sondereregger T.B. (1985). Women and weight: A normative discontent. Psychology and Gender.

[64] Gilbert P. (2014). The origins and nature of compassion focused therapy. Br. J. Clin. Psychol..

[65] Lillis J., Hayes S.C., Bunting K., Masuda A. (2009). Teaching acceptance and mindfulness to improve the lives of the obese: A preliminary test of a theoretical model. Ann. Behav. Med..

[66] De Graaff A.A., Dirksen C.D., Simoens S., De Bie B., Hummelshoj L., D’Hooghe T.M., Dunselman G.A.J. (2015). Quality of life outcomes in women with endometriosis are highly influenced by recruitment strategies. Hum. Reprod..

